# Ovarian adenocarcinoma in a young female with skin and umbilical metastasis

**DOI:** 10.4103/0971-5851.65339

**Published:** 2009

**Authors:** Pragya Shukla, Deepak Gupta, Shyam S. Bisht, M. C. Pant

**Affiliations:** *Department of Radiotherapy, Chatrapati Shahuji Maharaj Medical University, Lucknow, Uttar Pradesh – 226 000*

**Keywords:** *Ovarian carcinoma*, *skin metastasis*, *umbilical metastasis*

## Abstract

We report the case of ovarian carcinoma with skin and umbilical metastasis in a 30-year-old female. The computed tomography (CT) scan of the abdomen showed a right ovarian mass with anterior abdominal wall metastasis. The CT-guided fine needle aspiration cytology (FNAC) from the ovarian mass showed adenocarcinoma. FNAC from the umbilical and skin metastasis also showed adenocarcinoma. Because of the unresectability of the mass, the patient was put on taxol-based chemotherapy, which she took for two cycles, and then died of progressive disease after three months.

## INTRODUCTION

Ovarian cancer, a disease of ‘older women’ occurs primarily in postmenopausal women, with a peak incidence from 50 through 70 years of age. The disease is relatively uncommon before 40 years of age. Metastasis occurs primarily through the lymphatic and hematogenous pathways. A metastatic malignancy of the umbilicus commonly termed ‘Sister Mary Joseph nodule’ (SMJN) is a rare occurrence. Metastasis to the skin is still rarer, the reported incidence being 3%.[1] We report a cytologically proven case of adenocarcinoma of the ovary in a young female with skin and umbilical metastasis.

## CASE REPORT

A 30-year-old female presented to our Outpatient Department (OPD) in October 2007, with a history of vague gastrointestinal complaints of dyspepsia, nausea, and constipation for the past three months, and a progressively increasing abdominal swelling for the past one month. She had developed a nodule in the epigastric area ten days back and another nodule in the periumbilical area a week back.

**Figure 1:**
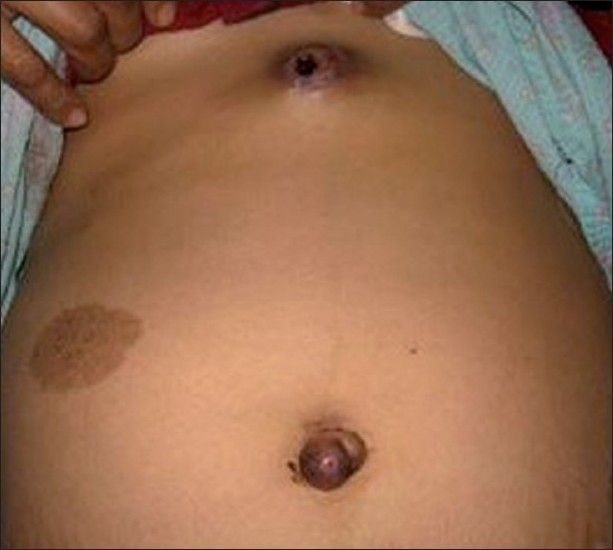
Photograph of patient showing skin and umbilical metastasis

On examination, the abdomen was distended and tense. Fluid thrill could be elicited. Bilateral inguinal lymphadenopathy was present. There was presence of umbilical hernia along with a nodule present over it discharging a serosanguinous fluid. An ulcerated nodule discharging a serosanguinous fluid was present in the epigastric area also [Figure 1].

**Figure 2:**
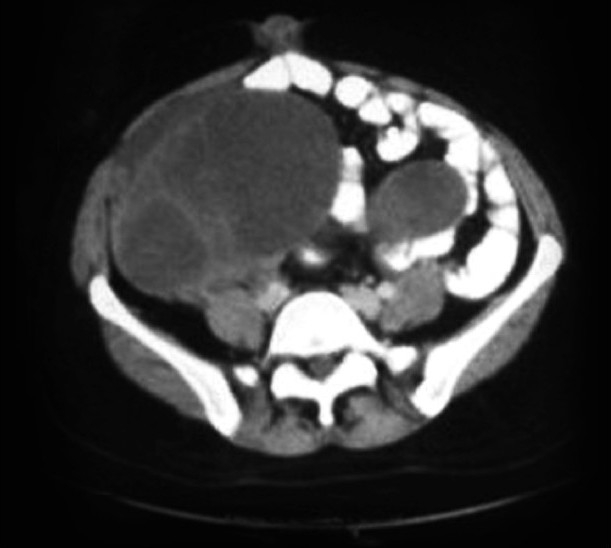
CT scan of the abdomen showing adnexal mass and skin metastasis

The CT scan of the abdomen [Figure 2] showed very large, bilateral, solid-cystic, heterogeneously enhancing abdominopelvic lesions extending from the bilateral adnexal regions to the abdomen. These lesions were abutting-involving the fundus and posterior wall of the uterus and also abutting the superior part of the urinary bladder with focal loss of intervening fat planes.

Multiple small-to-medium sized hypodense heterogeneously enhancing lesions were seen in both lobes of the liver. Anterior abdominal wall deposits were present. There was a presence of retroperitoneal, diaphragmatic, and bilateral inguinal lymphadenopathy along with the ascites.

**Figure 3:**
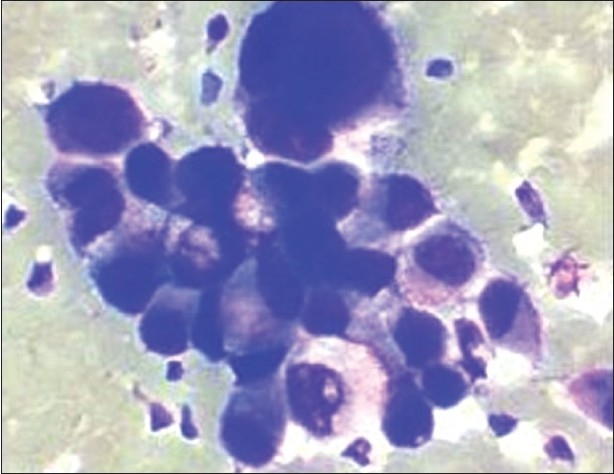
FNAC from ovarian mass showing marked pleomorphism and prominent nucleoli

CT-guided FNAC from the right ovarian solid cystic mass showed loosely cohesive ball-like clusters, acini and singly dispersed atypical cells on a proteinaceous background. Some of the clusters showed papillary disposition. The tumor cells displayed marked pleomorphism, high nucleocytoplasmic ratio, scant-to-moderate amount of pale cytoplasm, ill-defined cell borders, round-to-irregular nuclei, open chromatin, and prominent nucleoli. Mucin was present extracellularly as well as intracellularly. Binucleated and bizarre tumor cells were also present. The above-mentioned features were indicative of an adenocarcinoma of the ovary [Figure 3].

**Figure 4:**
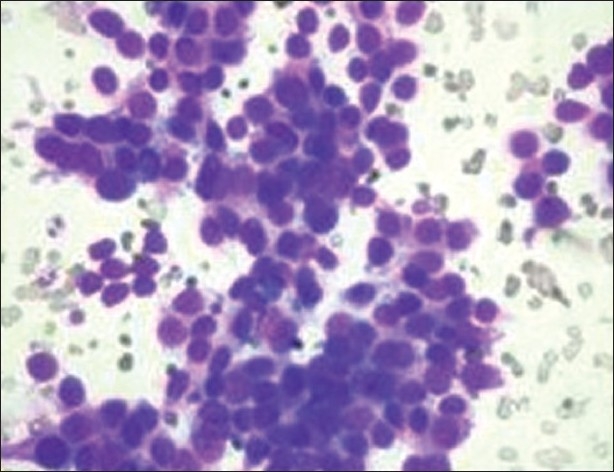
FNAC from skin and umbilical nodule showing pleomorphic cells with a high nuclear cytoplasmic ratio and hyperchromatic nucleus

FNAC from the skin and umbilical nodule showed atypical cells disposed in discohesive sheets and clusters, having a morphology similar to the primary ovarian adenocarcinoma [Figure 4]. Her CA-125 was 377.9 u/ml, and X-ray of the chest was normal.

Surgical resection was not possible due to the extensive local spread. In view of the fact that the primary tumor was not resectable and skin metastases were present, the patient was put on chemotherapy. She was planned for Taxol-based chemotherapy, which she took for two cycles and then died of progressive disease after three months.

We report this case as a rarity of occurrence of adenocarcinoma ovary in a young female and also for the presence of skin and umbilical metastases.

## DISCUSSION

A periumblical nodule (Sister Mary Joseph nodule) is mostly suggestive of an abdominal malignancy. In about 75% of the cases, the histological type is adenocarcinoma, and is very rarely epidermoid, undifferentiated or carcinoid. In over 55% of the cases, the origin is from the digestive tract. A case report describing SMJN with a gastric primary has been recently published.[2] Andreas Larentzakis *et al*. had reported three cases of SMJN, with the primary tumors being, adenocarcinona of the sigmoid colon, carcinoma of the bladder, and adenocarcinoma of the gallbladder, respectively.[3] A gynecological origin is the second most common etiology, with ovarian cancer being the most common (34% of the cases).[4,5] This is usually associated with poor prognosis. Average survival after discovery is about 10 to 11 months.[6] The interval time between diagnosis of ovarian cancer and documentation of cutaneous involvement is the most important prognostic factor associated with survival.[7]
